# Genome-Wide Association Study of Rice Rooting Ability at the Seedling Stage

**DOI:** 10.1186/s12284-020-00420-5

**Published:** 2020-08-24

**Authors:** Xin Xu, Junhua Ye, Yingying Yang, Mengchen Zhang, Qun Xu, Yue Feng, Xiaoping Yuan, Hanyong Yu, Yiping Wang, Yaolong Yang, Xinghua Wei

**Affiliations:** State Key Laboratory of Rice Biology, China National Rice Research Institute, Hangzhou, China

**Keywords:** Rice, Root growth ability, GWAS, Genetic architecture

## Abstract

**Background:**

Rice rooting ability is a complex agronomical trait that displays heterosis and plays an important role in rice growth and production. Only a few quantitative trait loci (QTLs) have been identified by bi-parental population. More genes or QTLs are required to dissect the genetic architecture of rice rooting ability.

**Results:**

To characterize the genetic basis for rice rooting ability, we used a natural rice population, genotyped by a 90 K single nucleotide polymorphism (SNP) array, to identify the loci associated with rooting-related traits through the genome-wide association study (GWAS). Population structure analysis divided the natural population into two subgroups: *indica* and *japonica*. We measured four traits for evaluating rice rooting ability, namely root growth ability (RGA), maximum root length (MRL), root length (RL), and root number (RN). Using the association study in three panels consisting of one for the full population, one for *indica*, and one for *japonica*, 24 SNPs associated with rooting ability-related traits were identified. Through comparison of the relative expression levels and DNA sequences between germplasm with extreme phenotypes, results showed that *LOC_Os05g11810* had non-synonymous variations at the coding region, which may cause differences in root number, and that the expression levels of *LOC_Os04g09900* and *LOC_Os04g10060* are closely associated with root length variation.

**Conclusions:**

Through evaluation of the rice rooting ability-related traits and the association mapping, we provided useful information for understanding the genetic basis of rice rooting ability and also identified some candidate genes and molecular markers for rice root breeding.

## Background

Rice is the staple food for most regions in Asia and feeds more than half of the world’s population (Hoang et al. 2019; Jiang et al. 2019). Rice production faces challenges posed by decreasing cultivated land and water resources. The root system of rice plays a key role in absorbing nutrients and water. Rice root is also an important synthetic organ of some metabolites and closely related to above-ground agronomical traits (Liang et al. 2011). Rice with a vigorous root system has greater tolerance to stress (Mahender et al. 2015) and is better equipped to compete with weeds (Anandan et al. 2016).

Most research on rice root has concentrated on investigating seedling root morphological traits and physiological and biochemical indexes under different stress conditions (Rohila et al. 2019). We classified the cloned root-related genes into three categories based on function. The first category included the genes that regulate root system morphological characters, such as *ARL1*/*CRL1*, *SRT5*/ *Oscyt-inv1*, *OsGNA1* and *WOX11* (Inukai et al. 2005; Liu et al. 2005). *Crown rootless 1* (*crl1/ARL1*) encodes a protein with a LATERAL ORGAN BUONDARIES (LOB) domain, which is involved in auxin-mediated cell dedifferentiation and controlled the initiation of adventitious root primordia in rice. *SHORT-ROOT5* (*SRT5*/ *Oscyt-inv1*) encodes a putative cytosolic Inv-N that cleaves sucrose at pH 7.0 ~ pH 8.0 and is the key isoform of Inv-Ns required for carbon and energy supply during early root development (Yao et al. 2009). The *Oscyt-inv1* mutant had an accumulation of sucrose but reduction of hexose. The cell length along the longitudinal axis of the root was reduced, and the cell shape in the root elongation zone shrank (Jia et al. 2008). *OsGNA1* encodes a Glucosamine-6-Phosphate acetyltransferase that is involved in de novo UDP-N-acetylglucosamine biosynthesis and influences the cell metabolism, microtube stabilization, and cell shape in rice roots (Jiang et al. 2005). *WOX11*, a WUSCHEL-related homeobox gene, is involved in the activation of the crown root emergence and growth. It can interact with the AP2/ERF protein, *ERF3*, which is involved in auxin-and cytokinin-responsive gene expression. The two genes function cooperatively to influence the crown root development by regulating the gene expression involved in cytokinin signaling (Chen et al. 2015; Zhao et al. 2009; Zhao et al. 2015). The second category of cloned genes was genes related to abiotic stress tolerance, like *OsP5CS* and *OsNAC9. OsP5CS* encodes a △^1^-pyrroline-5-carboxylate synthetase and plays an important role in proline accumulation under salt stress. Overexpression *OsP5CS* transgenic plants showed a better root growth (Zhu et al. 1998). STRESS-RESPONSIVE NAC 1 *(SNAC1/OsNAC9*) encodes a novel NAC-domain transcription factor and overexpressing this gene significantly enhances drought resistance in transgenic rice and transgenic lines exhibited altered root architecture involving an enlarged stele and aerenchyma (Redillas et al. 2012). The third category of genes participates in the absorption and utilization of mineral elements, such as *OsPTF1*. *OsPTF1* (*rice Pi starvation-induced transcription factor 1*) encodes a novel transcription factor with helix-loop-helix domain. Overexpression of *OsPTF1* transgenic rice plants showed significantly higher total root length and root surface area and enhanced the tolerance to Pi starvation in rice.

Little research has been done on gene clones related to rice rooting ability. Rice rooting ability means the ability of rice to generate new roots. It depends on the number of root primordia on the stem nodes of the seedling and the nutrients provided by the plant (Dai et al. 2008). Rice rooting ability differs among rice varieties and is influenced by heterosis (Hu et al. 2004; Zheng et al. 1996). New root occurrence rating is a key factor in rice tillering (Dai et al. 2008). Plants that produce more new roots have an obvious root vigor advantage (Dai et al. 2001; Ren et al. 2007). Root growth ability and root number were confirmed to significant positively correlate with seed setting rate and 2,3,5-triphenyltetrazolium chloride (TTC) reduction ability of rice at booting stage (He et al. 2006).

Genome-wide association studies (GWAS) have been used successfully in rice to analyze the genetic basis of agronomic traits. Based on phenotype evaluation and genotyping, GWAS is an effective way to isolate the gene related to the target trait. For instance, researches using GWAS found that *OsSPL13* (Si et al. 2016) controls the grain length, *GSE5* (Duan et al. 2017) controls the grain width, the *bsr-d1* allele is associated with Digu Blast resistance (Li et al. 2017), *qPSR10* (Xiao et al. 2018) is related to cold tolerance, and *qTIPS-11* is associated with lateral root number (Wang et al. 2018). Recently, *OsSPY* (Yano et al. 2019), which is associated with semi-dwarfism and small panicles, was identified by GWAS with principal component analysis.

In our research, 145 accessions were used to conduct the GWAS. The goals were (1) to assess the natural variation for the traits related to rice seedling rooting ability in the diverse rice panel, (2) to identify the loci and candidate genes related to rice rooting ability, and (3) to find varieties with superior rooting ability to be used in rice breeding.

## Results

### Population Divergence and Relative Kinship Analysis of the Rice Accessions

**Fig. 1:**
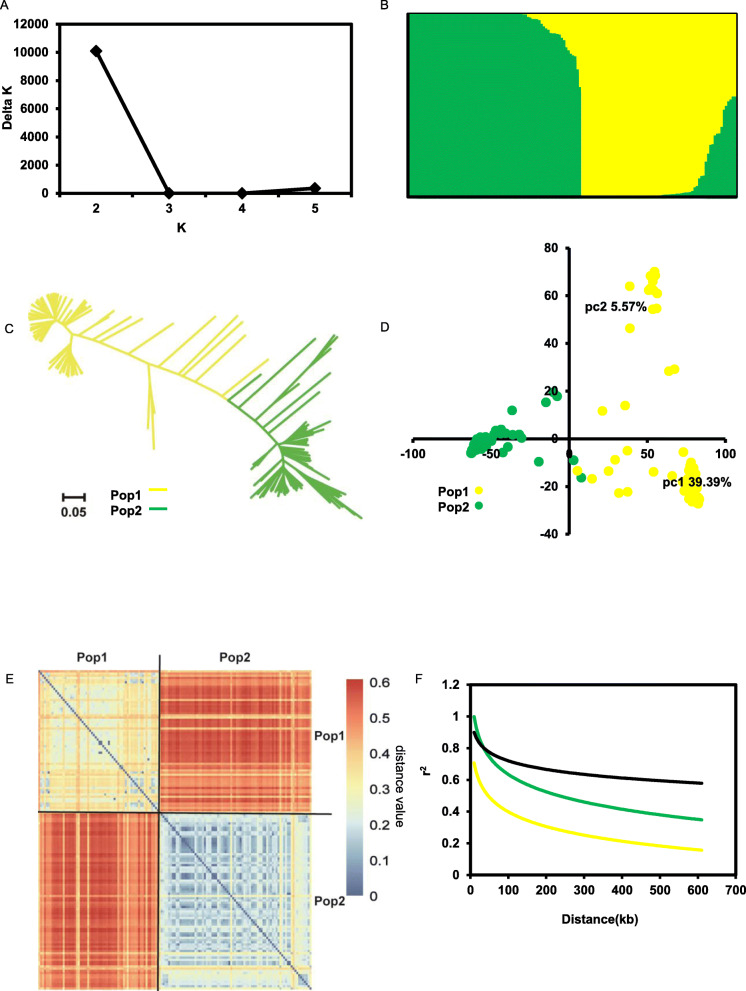
Population structure of the 145 rice accessions. **a** Delta K values plotted as the number of subpopulations. **b** Subpopulations (K = 2) inferred using STRUCTURE. **c** NJ tree based on Nei’s genetic distances. Yellow and green represent Pop1 and Pop2, respectively. **d** Principal component analysis of rice panel. Values in parentheses indicate the percentage of variance in the data explained by each principal component. **e** Pairwise relative kinship analysis by distance matrix of rice panel. **f** Genome-wide average LD decay estimated from 145/full (black), 56 Pop1/*indica* (yellow) and 89 Pop2 / *japonica* (green) landraces

Total 145 rice accessions were used in our research, most of which were collected in southern regions of China. About 65 K SNP genotype of these 145 accessions was obtained from a 90 K high-density SNP array. After filtering the SNPs with minor allele frequency less than 0.05 or a minimum count less than 75% of 145 individuals by TASSEL 5.2.51 (Bradbury et al. 2007), 56,456 SNPs distributed on all 12 chromosomes were used for the final genetic analysis. Only about 0.3% of inter-SNP distances were greater than 50 kb (Additional files 1: Figure S1A). Based on the nucleotide polymorphism, we first calculated the genetic component of each variety. The results showed that the value of Evanno’s ΔK had the highest value at K = 2 (Fig. 1a). Therefore, combined with varieties’ original information, we speculated that two subspecies, *indica* (Pop1) and *japonica* (Pop2), were present in our natural population (Fig. 1b). The same results could also be observed from the neighbor joining (NJ) tree (Fig. 1c). Similarly, principle component analysis (PCA) results distinguished the accessions into two subpopulations, PC1 and PC2, which accounted for 39.39% and 5.57% of the genetic variation, respectively (Fig. 1d). Together, results of kinship relatedness of the pairwise accessions showed that a few accessions within Pop2 had relatively strong relatedness, while the kinship relatedness among the accessions in the Pop1 was weak (Fig. 1e). Similar results were observed from the NJ tree, in which the accessions in Pop2 showed stronger kinship relatedness than the accessions in Pop1. Moreover, the population differentiation statistics (*F*_*ST*_) value between the two subpopulations was 0.71, indicating that a high level of subpopulation differentiation existed in the 145 accessions. Finally, we analyzed the linkage disequilibrium (LD) rate for three populations using the SNP data. The genome-wide LD decay rates of Pop1, Pop2, and the full population were estimated to be about 200 kb, 300 kb, and 600 kb, respectively (Fig. 1f). These results showed that the density of the SNP used in our study is sufficient for the GWAS.

### Evaluation of the Rooting Ability of Rice

**Fig. 2:**
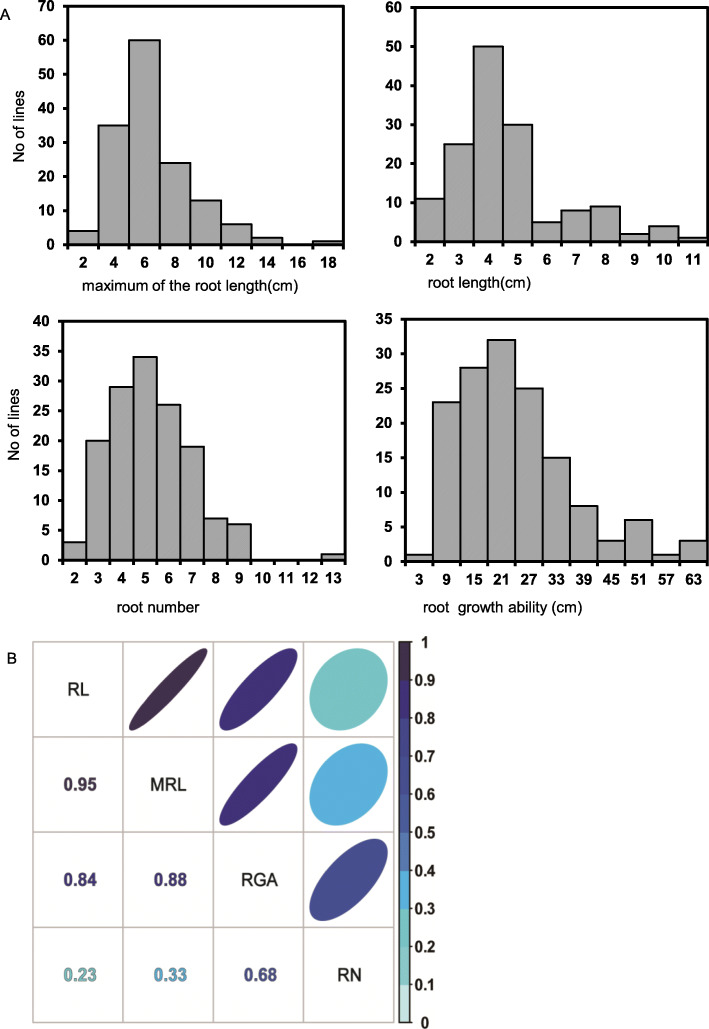
Phenotypic diversity of the 145 rice accessions. **a** Phenotypic distribution of root-related traits in the natural population. **b** Correlation analysis of root-related traits. The color and area of the circle represent the value of the correlation coefficient. The number in the middle of the cell is the correlation coefficient

In our research, four rooting-related traits were measured at the seedling stage to evaluate rice rooting ability. These four traits root growth ability (RGA), maximum root length (MRL), root length (RL), and root number (RN) were all close to normal distribution and had abundant variations (Fig. 2a, Additional files 1: Table S1). Among them, RGA had the highest coefficient of variation value (CV = 59.97%), followed by MRL, RL, and RN, and all of them had a CV value larger than 30% (Table 1). RN had the highest H′ value of diversity index at 0.41 (Table 1), followed by RGA (0.39), MRL (0.38), and RL (0.37). These results indicated that our natural population had abundant phenotypic variations. Correlation analysis results revealed that RGA had a high positive correlation with the other three traits (Fig. 2b). The correlation coefficient between MRL and RL was the highest (*r* = 0.95). RN was moderately correlated with RGA (*r* = 0.68) and weakly correlated with RL and MRL. These results may indicate that RN is a relatively independent trait for evaluating rooting ability compared with the other traits in our research. The four traits’ phenotypic variation explained by population structure (*R*_Q_^2^) were significant (*p <* 0.01), but all *R*_Q_^2^ values were relatively low. The RGA had the lowest *R*_Q_^2^ value at 0.08 (Table 1). In addition, the comparison of the mean value of the four traits between the two subgroups (Pop1 and Pop2) by one-way analysis of variance (ANOVA) revealed obvious differences in MRL (*p* = 0.0436), RL (*p* = 0.0118), and RN (*p* = 0.0133), with the exception of RGA (*p* = 0.9266) (Additional files 1: Figure S1B). These results suggest that the effect of population structure should be considered into the next GWAS analysis.

**Table 1:** phenotypic variation for the root growth ability and related traits in 145 rice accessions

trait	mean ± SD	range	CV	diversity of H′ value	R_Q_^2^
MRL	5.59 ± 2.63	1.50 ~ 17.77	47.05%	0.38	3.73%
RL	4.17 ± 1.89	1.12 ~ 10.36	45.32%	0.37	5.76%
RN	4.85 ± 1.70	1.56 ~ 12.22	35.05%	0.41	4.48%
RGA	20.76 ± 12.45	2.34 ~ 60.47	59.97%	0.39	0.08%

### GWAS Analysis Results for Rooting Ability Traits in Rice

**Fig. 3:**
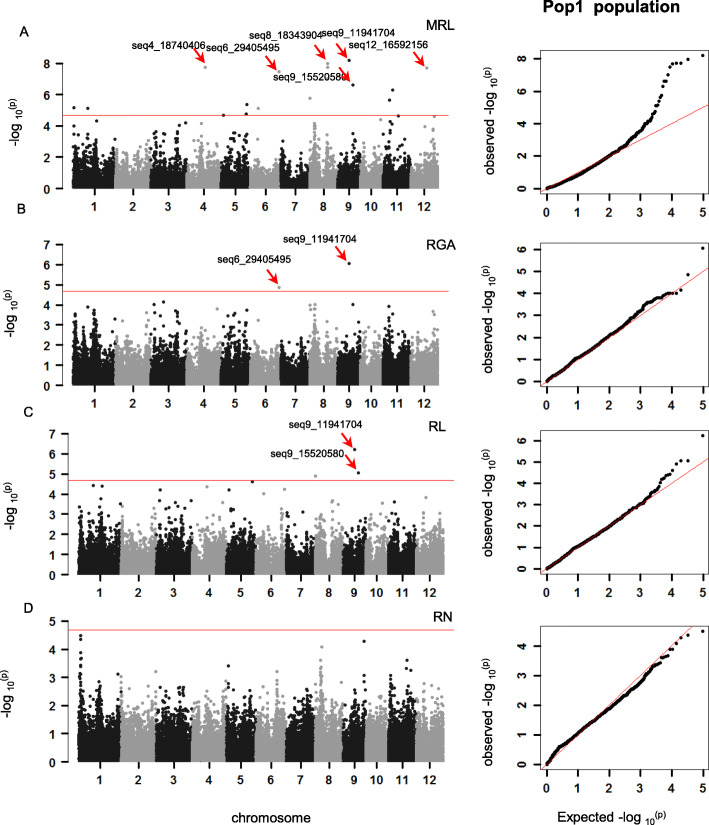
GWAS for rooting ability–related traits in the Pop1 association panel. Manhattan plots and quantile-quantile plots for MRL (**a**), RGA (**b**), RL (**c**), RN (**d**). The red line in the manhattan plot represents the significance threshold. The red straight line in the quantile-quantile plot represents expected null distribution of *p*-value and black dots represent observed distribution of *p*-values. MRL: maximum root length, RGA: root growth ability, RL: root length, RN: root number

**Fig. 4:**
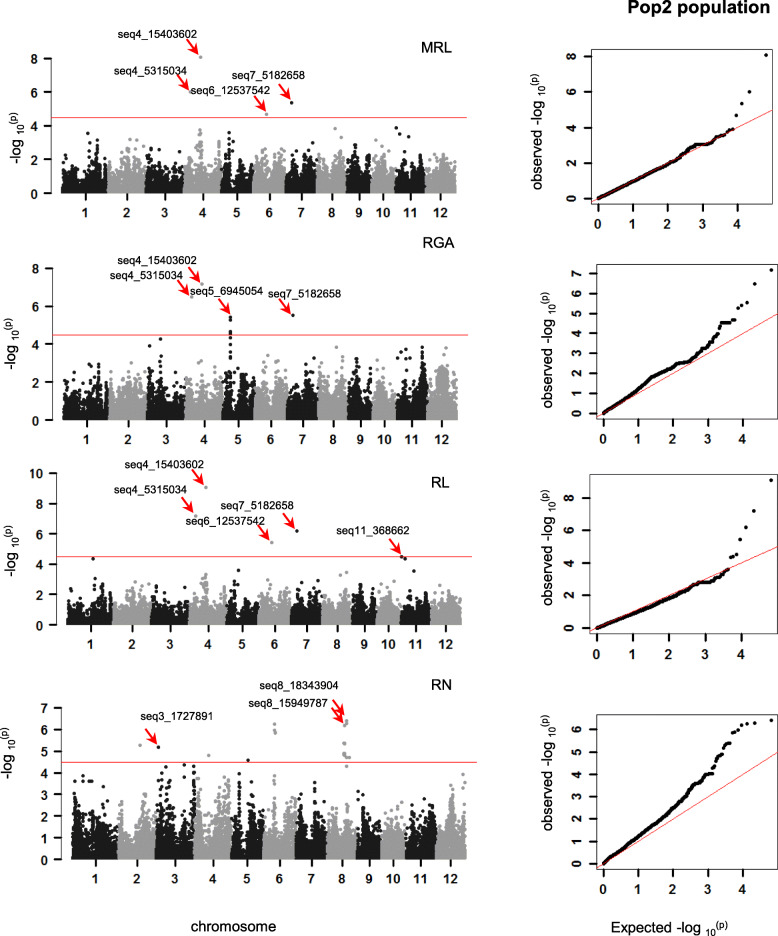
GWAS for rooting ability–related traits in the Pop2 association panel. Manhattan plots and quantile-quantile plots for MRL (**a**), RGA (**b**), RL (**c**), and RN (**d**)

Association mapping was conducted in three association panels of the full, Pop1, and Pop2 using the different models to minimize the impact of the population structure on the power of GWAS as well as to avoid the over-correcting (Additional files 1: Figure S2). In total, 38 (*p* < 1.8 × 10^− 5^), 23 (*p* < 2.1 × 10^− 5^), and 43 (*p* < 3.2 × 10^− 5^) suggestive SNPs were detected in the full (Additional files 1: Figure S3), Pop1 (Fig. 3), and Pop2 (Fig. 4) association panels for the four traits, respectively. These SNPs were distributed on all chromosomes with the exception of chromosome 10 and 2. Moreover, it had several SNP hotspots (Additional files 1: Figure S4).

Mixed linear model (MLM) was used for GWAS in the full population. MRL-related SNPs were detected most commonly (Additional files2: Table S2), followed by RL, RGA, and RN. The SNP seq4_15403602 had the lowest *p*-value and the highest explanation rate at 31.62%, 30.22%, and 27.03% for the RL, MRL and RGA, respectively. Additionally, there was one region contained the cluster SNPs which was correlated with RGA. And the lead SNP seq5_6945054 had the lowest *p*-value and could explain 14.14% RGA variation.

A total of 23 significant SNPs were detected in the Pop1, and most of them were related to MRL, followed by RL and RGA. No significant SNP was detected for RN (Additional files 2: Table S3). The important SNP seq9_11941704 had the lowest *p*-value for RL, MRL, and RGA and explained 39.33%, 47.56%, and 37.50% of phenotypic variation, respectively. Only one region containing clustering SNPs was detected for MRL, and the leading SNP seq8_18343904 could explain 43.66% of MRL variation.

In the Pop2 panel, the number of SNPs detected for RN was the highest, followed by RGA, RL, and MRL (Additional files 2: Table S4). The SNP seq4_15403602, which had been also detected in the full panel with the lowest *p*-value, had the highest phenotypic explanation rate for RL, MRL, and RGA in this panel. The SNP seq8_18343904 with the lowest *p*-value for RN could explain 30.20% phenotypic variation of it, and this SNP was also identified in Pop1 for MRL. In addition, there were another three regions containing the clustering SNPs with the lead SNPs seq6_1153742, seq8_15949787, and seq5_6945054, where seq6_1153742 and seq8_15949787 explained 28.15% and 32.07% of the RN variation rate, respectively, and seq5_6945054 explained 23.89% of RGA variation, which could also be detected in the full panel.

### Identifying the Candidate Genes Associated with the Rooting Ability of Rice

To obtain candidate regions from these significant SNPs, we combined the suggestively significant SNPs with quantile-quantile (Q-Q) plots of the GWAS results. We then focused on the SNPs distributed in clusters and detected by more than one panel to avoid false positives if the Q-Q plot was not well-fitted. Based on this, a total of 24 SNPs (Table 2) were obtained.

**Table 2:** The 24 significant SNPs and their rice genomic information

Trait	marker	population-identyfied	allele	Chr	Pos	*p*-value	candidate genes	functional annotations
RN	seq3_1727891	full, Pop2	C/T	3	1727891	6.54E-06	*CDPK13* (Ho et al., 2013)	gibberellin biosynthesis
								drought stress
MRL,RGA,RL	seq4_5315034	full, Pop2	A/G	4	5315034	9.69E-07	*OsCyc1* (Otomo et al., 2004);	allelopathy
							*OsDTS2* (Wilderman et al., 2004)	allelopathy
MRL,RGA,RL	seq4_15403602	full, Pop2	T/C	4	15403602	8.52E-10		
MRL	seq4_18740406	Pop1	C/G	4	18740406	1.84E-08	*SLL1/OsSAD5* (Shelley et al., 2013)	lateral root length
								,fatty acid denatures
RGA	seq5_6882162	Pop2	T/C	5	6882162	2.88E-05	*OsPYL* (Kim et al., 2014);	abscisic acid receptor
							*OsGA2ox10* (Lo et al., 2008)	gibberellin 2-oxidases
RGA	seq5_6884530	Pop2	C/A	5	6884530	2.88E-05		
RGA	seq5_6890004	Pop2	G/A	5	6890004	2.88E-05		
RGA	seq5_6908893	Pop2	T/C	5	6908893	2.88E-05		
RGA	seq5_6935074	Pop2	T/G	5	6935074	2.88E-05		
RGA	seq5_6945,054	full, Pop2	T/C	5	6945054	5.54E-06		
RGA	seq5_6959356	Pop2	G/A	5	6959356	2.88E-05		
RGA	seq5_6981293	Pop2	T/C	5	6981293	2.88E-05		
MRL,RL	seq6_12537542	full, Pop2	T/C	6	12537542	3.73E-06	*OsPT9* (Wang et al., 2014)	phosphate uptake
MRL,RGA	seq6_29405495	full, Pop1	C/T	6	29405495	3.30E-08	*OsMPK4* (Agrawal et al., 2003)	salicylic acid and jasmonic
								acid-signaling pathway
MRL,RL,RGA	seq7_5182658	full, Pop2	A/G	7	5182658	1.25E-08	*SQS* (Manavalan et al., 2012)	root length
								drought tolerance
MRL,RL	seq8_433198	full, Pop1	G/T	8	433198	1.25E-05		
RN	seq8_15949787	Pop2	G/C	8	15949787	6.51E-07	qtl (Lilley et al., 1996)	dehydration tolerance
MRL,RN	seq8_18343904	Pop1,Pop2	T/G	8	18343904	1.05E-08		
MRL,RN	seq8_18364190	full, Pop1,Pop2	C/T	8	18364190	1.77E-08		
MRL	seq8_18365309	Pop1	C/T	8	18365309	1.00E-07		
MRL,RL,RGA	seq9_11941704	Pop1	T/C	9	11941704	6.28E-09		
MRL,RL	seq9_15520580	Pop1	A/C	9	15520580	2.45E-07	*OsGL1–1* (Islam et al., 2009)	drought tolerance
MRL,RL	seq11_368662	full, Pop2	A/G	11	368662	5.23E-06		
MRL	seq12_16592156	Pop1	T/C	12	16592156	2.00E-08	*DEC* (Itoh et al. 2012)	cytokinin signaling pathway root length

Traditionally speaking, the gene nearest to SNP is not always the causal gene. Therefore, we screened the candidate genes from the LD blocks mentioned according to their annotation information from the China Rice Data Center (http://www.ricedata.cn/gene/) and the Rice Genome Annotation Project (http://rice.plantbiology.msu.edu/index.shtml). We mainly selected genes with GO information related to hormone metabolism processes or stress responses or genes which have the high expression level at seedling stage. As a result, some known genes and QTLs reported previously were obtained (Table 2). By summarizing the given annotation information, these genes and QTLs could be classified into four categories. The first category included three genes related to endogenous hormone signaling pathway. A calcium-dependent protein kinase gene (*OsCDPK13*) was located at about 99 kb upstream of the SNP seq3_1727891. It has been found to negatively regulate the expression level of the enzymes essential for gibberellin biosynthesis and prevent drought stress injuries in the seedling development stage (Ho et al. 2013). The mitogen-activated protein kinase gene (*OsMPK4*) was mapped at about 4 kb upstream of the SNP seq6_29405495. Its transcript’s level is up-regulated upon wounding (by cut), hormones, and heavy metals, et al. (Agrawal et al. 2003). *DECUSSATE* gene (*DEC*) participated in the cytokine signaling pathway of rice, and seminal and crown roots of the mutant were short and thin (Itoh et al. 2012). In the second category, one gene was related to phosphate uptake. A phosphate transporter gene (*OsPT9*) was identified at about 142 kb downstream of the SNP seq6_12537542. Its expression level is specifically induced by Pi starvation (Wang et al. 2014). In the third category, one gene was responsible for root drought tolerance. *Glossy 1*(*OsGL1–1*) was located at 24 kb upstream of the nearest SNP seq9_15520580. And *osgl1–1* mutants showed increased sensitivity to drought (Islam et al. 2009). In the fourth category, two genes were correlated with morphogenesis of roots. *Farnesyltransferase/squalene synthase* (*SQS*) and *SHORT LATERAL ROOT LENGTH1* (*SLL1*) directly influence root architecture and were found at 257 kb downstream of the SNP seq7_5182658 and 348 kb upstream of the SNP seq4_18740406, respectively. RNAi of the *SQS* plants grown in plates with Yoshida’s nutrient solution with 1.2% agar showed increased root length and an enhanced number of lateral roots (Manavalan et al. 2012). *SLL1* encodes stearoyl-acyl carrier protein and over-expression *SLL1* plants produced significantly longer lateral roots compared to wild-type plants (Shelley et al. 2013). Meanwhile, one region containing the lead SNP seq8_15949787 belongs to a previously reported QTL (Lilley et al. 1996) of root dehydration tolerance.

In total, 26 genes were used to conduct the quantitative real-time PCR (qRT-PCR). The primers used for the qRT-PCR are listed in the supplementary materials (Additional files 2: Table S5). Considering these loci responsible for different traits and the correlation among them, we mainly chose varieties with contrasting RN or RL. Taking into consideration that these two traits exhibit differences between the *indica* and *japonica* varieties, we mainly chose the varieties within the same subpopulation. More importantly, taking into consideration the fact that a few genes had low expression levels in roots and proved to be outstanding candidate genes for improved abiotic stress resistance, we also sequenced possible genes in varieties with contrasting phenotypes to find possible excellent haplotypes. Interestingly, we not only found two genes that differ in relative expression level between differing RL (Additional files 2: Table S6), but we also found that one gene’s sequence variation is associated with RN. The results are as follows.

**Fig. 5:**
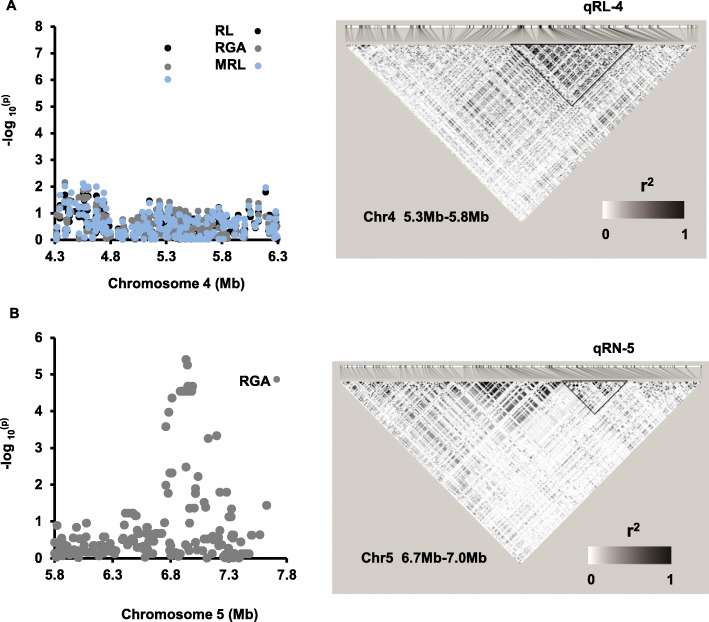
Estimation of the candidate regions by local linkage disequilibrium analysis on chromosome 4 and chromosome 5. **a** Local manhattan plots (left) of GWAS for MRL, RL, and RGA for the Pop2 association panel and linkage disequilibrium heatmap (right) surrounding the peak on Chromosome 4. **b** Local manhattan plots (left) of GWAS for RGA for the Pop2 association panel and linkage disequilibrium heatmap (right) surrounding the peak on Chromosome 5

**Fig. 6:**
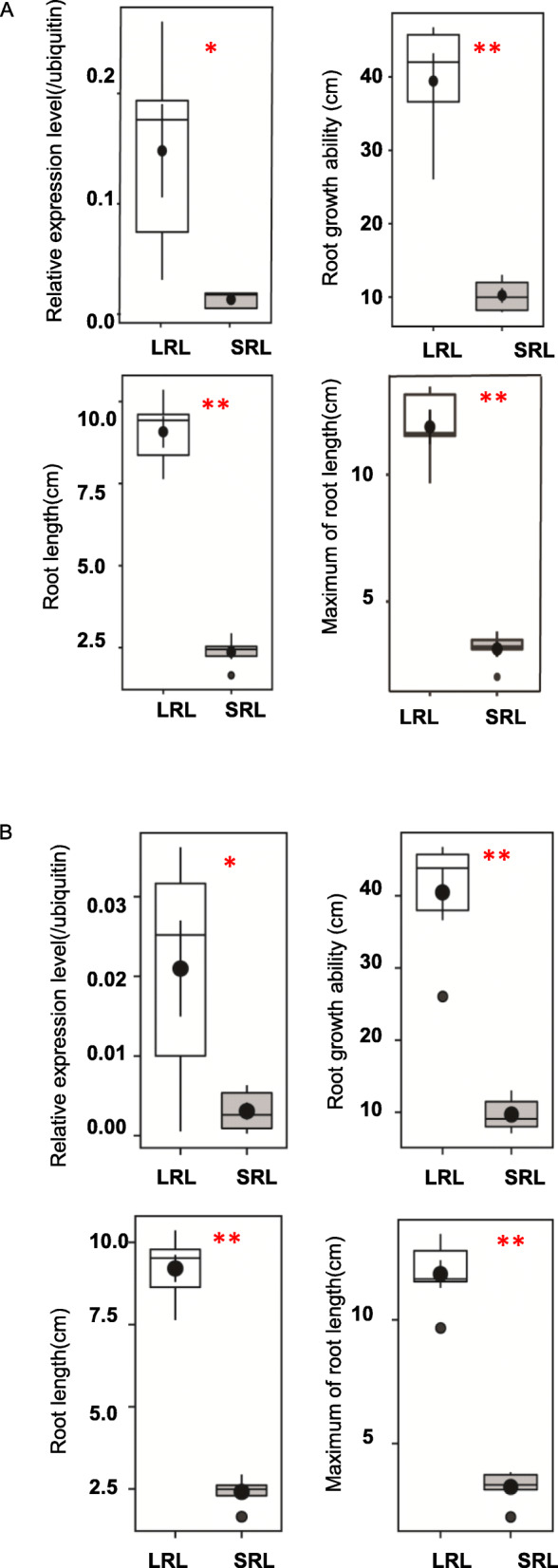
Comparison of the relative expression level of candidate genes between groups with contrasting phenotypes in qRL-4. **a** Boxplots of relative expression level, RN, MRL, and RGA in different groups with contrasting phenotypes of *LOC_Os04g09900* (*OsCyc1*). LRL means varieties in the group with longer root length and SRL means the varieties in the group with shorter root length. Box edges represent the 0.25 and 0.75 quantiles with the median values shown by bold lines. Whiskers extend to data no more than 1.5 times the interquartile range and the remaining data are indicated by asterisks. “*” on the top of the boxplots represents the significance at 0.05 probability levels and “**“represents the significance at 0.01 probability levels. **b** Boxplots of relative expression level, RN, MRL, and RGA in different groups with contrasting phenotypes of *LOC_Os04g10060* (*OsDTS2*)

**Fig. 7:**
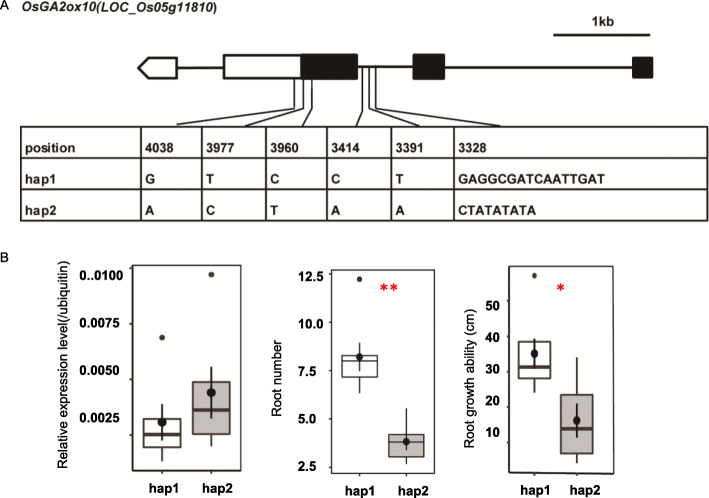
Analysis of DNA polymorphisms in the *OsGA2ox10* gene and comparison of relative expression level of it between groups with different haplotypes. **a** Gene structure of the candidate gene *LOC_Os05g11810* (*OsGA2ox10*); the solid box and empty box indicate the exon and untranslated region, respectively. **b** Boxplots of relative expression level, RN and RGA in different haplotypes of *LOC_Os05g11810* (*OsGA2ox10*)

One candidate region *qRL-4* of about 480 kb for RL, MRL, and RGA including the significant SNP is located at about 5.3 Mb on chromosome 4 (Fig. 5 A). In *qRL-4*, we determined that *LOC_Os04g09900* encodes syn-copalyl diphosphate (syn-CDP) synthase (OsCyc1) protein, which is responsible for phytoalexin biosynthesis (Otomo et al. 2004). A comparison of expression levels of this gene among the 10 accessions with extreme RL differences (*p* < 0.01) was conducted by qRT-PCR. *LOC_Os04g09900* had a higher expression level in the group with longer root length (LRL group) than the group containing varieties with shorter root length (SRL group) (*p* = 0.0330) (Fig. 6a). Another gene, *LOC_Os04g10060* (*OsDTS2*), is a syn-copalyl diphosphate specific 9β-pimara-7,15-diene synthase gene. Previous qRT-PCR results (Wilderman et al. 2004) showed that varieties owning longer root had higher expression level (Fig. 6b). Another region, *qRN-5*, for RN and RGA is about 300 kb. It contains a cluster of significant SNPs and the lead SNP is seq5_6945054 (Fig. 5b). Two known genes, *LOC_Os05g11810* (*OsGA2ox10*) and *LOC_Os05g12260* (*OsPYL*), were identified in *qRN-5*. *LOC_Os05g11810* encodes the gibberellin 2-oxidases (GA2oxs), which can regulate plant growth by inactivating endogenous bioactive gibberellin (GA) (Lo et al. 2008), and *LOC_Os05g12260* encodes a rice orthologue of the ABA receptor (Kim et al. 2014). From the sequencing results of the coding region, we only found sequence variations of *LOC_Os05g11810* among the varieties with root number differences (Fig. 7). Based on these, we divided varieties into two haplotypes (hap1 and hap2). Hap2, sharing the same haplotype with Nipponbare, had fewer roots compared with hap1 (*p* = 0.0005). Additionally, both two groups had low transcripts which may cause that although hap2 group had a higher mean value of relative expression level than hap1, it was not statistically significant (*p* = 0.3727).

## Discussion

### Characterization of the Rooting Ability in Rice Seedlings

A vigorous root system is the basis of vigorous growth in rice at early stage and grain filling at later stage. Furthermore, rooting ability was confirmed to be inherited and showed significant heterosis (Zheng et al. 1996). Rice rooting ability is determined by several parameters including seedling leaf age and seedling quality. Based on previous research, a nutrient solution was used for cultivating root-cut seedlings to provide a consistent nutritional environment to minimize the impact of the upper part on root systems. Simultaneously, we evaluated the rooting ability of rice at the seedling stage by measuring the morphological indicators of root system such as root length and root number. The four evaluated traits showed abundant variation and continuous distribution, suggesting that these traits related to rooting ability are quantitative traits and suitable for GWAS.

From the comparison of phenotypic traits in different populations, there were certain phenotypic differences between *indica* and *japonica* varieties. RN, RL, and MRL had differences at the significant level of *p* < 0.05 in the two subpopulations, while the difference of RGA between the two subpopulations was not significant. Similar results were also obtained from the analysis of the phenotypic variation explained by population structure, in which RGA have the lowest *R*_Q_^2^ value. This indicated that *japonica* tends to possess longer root length and *indica* tend to possess larger number of roots when their roots were cut at the seedling stage. This may be due to the different ecological environmental conditions of subpopulations. *Japonica* is generally distributed in temperate regions, high latitude regions, tropical and subtropical mountains, and arid regions. Rice plants in these regions require longer root length to absorb water. *Indica* is usually distributed in low latitudes and tropical and subtropical lowlands of China and Southeast Asia. The abundance of water in these regions may cause these varieties to have a higher number of relatively shallow roots and stronger tillering (Xu et al. 2012). This is consistent with the result of a previous study on rice roots (Zhao et al. 2019). There was no significant difference in the final RGA between the two subpopulations. It suggests that both two subpopulations possess considerable ability for rooting when suffering the root damage despite their different root morphology.

Correlation analysis showed that RGA is positively related with RL, MRL, and RN. By contrast, the correlation between RL and RN is weak. This may indicate that the genetic basis of RN is relatively independent from RL. These results corresponded to the association results, in which only one significant SNP could be detected for multiple traits.

Among these four evaluated traits, RGA had the highest CV value and most associated loci detected by GWAS. What’s more, there is no significant difference between the two subpopulations. So, we think RGA is an appropriate trait for evaluating the rice rooting growth ability. Additionally, RN is relatively independent, and difficult to be evaluated. For example, some varieties’ new roots are relatively short, while some new roots are long, and it is difficult to unify the criteria of a certain root length as one new growing root for calculating the number of new roots. And we select the root with the root length over 0.5 cm as one new root.

### GWAS Results Analysis among Different Association Panels

GWAS is an efficient way to analyze genetic variation for multiple traits in rice (Lu et al. 2016; Zhang et al. 2018). In GWAS, accuracy and precision are influenced by population structure, kinship, and LD decay rate.

In the present study, LD decay distance results showed that the full population had the highest LD decay distance. Similar results were reported in a previous study, suggesting that population mixing could introduce chromosomes from different ancestral sources and allele frequencies to affect LD level (Wang et al. 2007). Population structure analysis divided the full population into two distinct subpopulations. This result was supported by the results of PCA and the NJ tree. In addition, the distance matrix and NJ tree showed that only a few varieties in Pop2 have close kinship while the majority Pop1 varieties showed evidence of weak kinship relatedness. Thus, to avoid overcorrection, we used the MLM model for GWAS in the full population and GLM model in the two subpopulations. And Q-Q plots indicated the GWAS model used for each population is suitable. However, two subpopulations were small for the GWAS which may cause that loci with low effects are difficult to detect and only a few significant SNPs had been detected.

In our GWAS results, there were differences in the suggestive loci for different traits and different populations. Some SNPs were detected for more than one trait, which could be explained by pleiotropic genes. Most of significant SNPs related to RGA could also be detected in GWAS for RN or RL, and similarly, MRL and RL also shared the same SNPs discovered by GWAS. In contrast, there were SNPs identified in more than one GWAS panel. In most cases, the SNPs discovered by the full population were also could be detected by Pop1 or Pop2 panel. However, some SNPs could only be detected in the Pop1 or Pop2 association panel, which may be due to the low frequency of rare SNPs. As for some significant SNPs only detected in full association panel, which may be due to false positive caused by population structure. In addition, most loci related to RN were detected in *japonica*, while most loci related to MRL were detected in *indica*. This may be because *indica* and *japonica* have different domestication processes, leading to different phenotypic variations and different genetic backgrounds between the two subpopulations. There was only one candidate region including two SNPs detected simultaneously in the two subpopulations. It may reflect the considerable differences in the genetic background of the *indica* and *japonica* for rooting ability. The strong heterosis of rice rooting ability may be elucidated by the recombination of the positive loci in different subpopulations.

### Analysis of the Regulatory Mechanism of Participating in Rooting Ability

According to the GWAS results, the association study was efficient in determining the number of loci controlling rice rooting ability, enabling us to identify a series of cloned genes from the candidate regions. By summarizing the functions of these known genes, it suggested that hormone signaling pathways (*CDPK13*, *OsMPK4*and *DEC*), phosphorus absorption pathways (*OsPT9*), and the mechanism of both root morphology (*SQS* and *SLL1*) and drought-tolerance (*OsGL1–1*) may regulate rice rooting ability. These results may be illustrated by the fact that cutting roots causes nutrient and water deficiency and new roots are needed to absorb both nutrients and water. The results could also suggest that endogenous hormone metabolism could regulate the growth of new roots, which is consistent with previous research reported that the content of endogenous hormones in seedlings can affect the RGA of seedling rice. Specifically, endogenous hormone abscisic acid in roots can inhibit RGA, while endogenous hormone gibberellin can promote RGA (Ren et al. 2009). These results may provide us a perspective on rooting vigor breeding by aggregating the genes from these four pathways. However, further research will be necessary to confirm it.

### Identification of Candidate Genes Controlling the Root Growth Ability of Rice

Here, we identified three candidate genes that may be responsible for rice rooting. For the candidate genes identified by qRT-PCR analysis, both *OsCyc1* and *OsDTS2* had expression level differences for contrasting phenotype. *OsCyc1* is responsible for phytoalexin biosynthesis, and its transcript level increased after infection by rice blast (Otomo et al. 2004). *OsDTS2* (Wilderman et al. 2004) encodes a syn-copalyl diphosphate specific 9β-pimara-7,15-diene synthase. Its mRNA level in leaves is up-regulated by stimulating phytoalexin biosynthesis but is constitutively expressed in roots. Both of them had higher expression levels in groups with longer root length than in groups with shorter root length. Thus, we inferred that cutting roots caused abnormal growth and varieties with more transcripts could produce longer roots for absorbing water and nutrients. Furthermore, both genes are involved in allelopathy which was benefit for rice plants in suppressing growth of widespread rice paddy weeds. This is consistent with previous research that vigorous root systems in rice enhance competitiveness with weeds.

However, DNA sequence analysis showed that one gene’s sequence variation was closely associated with phenotype. We concluded that *OsGA2ox10* may be responsible for root number variation and the hap2 of the *OsGA2ox10* is the positive haplotype. In Lo’s research (Lo et al. 2008), *OsGA2ox10* was not detected the mRNA level, while the expression of the *OsGA2ox10* in our research was detected in roots, though the expression level was relatively low. Despite the relative expression and haplotype analysis verification, these three genes need to be verified using further genetic complementation experiments.

## Conclusion

In our study, we dissected the genetic basis of the rooting ability of rice at the seedling stage in *indica* and *japonica* rice subgroups. This provides valuable information for future study on the genetic basis of rice rooting ability. We also identified some candidate regions and genes which are related to rooting ability. Moreover, the SNPs found in these regions and genes could be used for future gene validation and marker-assisted selection. Our results may provide useful information for rice root breeding.

## Materials and Methods

### Plant Materials

A panel consisting of 145 accessions was used for GWAS. To ensure homogeneity, they were grown at the experimental farm of the China National Rice Research Institute in 2016 and 2017. Plant density was four lines per plot with six individuals per line. At harvest, the seeds were collected from the middle plants in each line.

### Phenotypic Evaluation at the Seedling Stage

After being fully air-dried, the seeds were soaked for 2 days in a germination-accelerating solution for 12 h at 30 °C. They were then sown in the soil at the green house. Three weeks later, the crown roots of each accession were cut off. The plants were then transferred into glass tubes filled with Yoshida nutrient solutions (Yoshida et al. 1971). Three single plants of each accession were fixed by black sponge in one glass tube for three replications in total. The glass tubes were surrounded by the black cloth to block direct light. Nutrient solutions were changed every 3 days. One week after root cutting, the number and length of new roots were counted and measured by ruler for three single plants per replication. Finally, four rooting-related traits were evaluated: RN, RL, MRL, and RGA. Three replicates were set for each variety, and RN was calculated by averaging the root number for all normal plants in three replications. The root length of healthy plants was measured in each replicate. Root length for one plant is defined as the mean value of the all new root length belonged to the plant and maximum root length for one plant is the root length of the longest root among the new roots. RL and MRL is the mean of root length and maximum root length of a variety’s all replicates, respectively. To find the RGA, RGA of a single plant was calculated using the formula: RGA = RN × RL (mean of the root length per plant). The average of the total single plant’s RGA for one accession was used as the final RGA for the GWAS analysis.

### Phenotypic Data Analysis

Mean value, standard deviation, and diversity index of the H′ value were all calculated in EXCEL 2010. The mean value and standard deviation were calculated using the AVERAGE () and STDEVP (). Based on the mean value and standard deviation of each trait, we divided 145 accessions into 10 levels, from the first level (Xi<X-2σ) to the tenth level (Xi>X + 2σ). Every 0.5σ was a level. According to these grading results, the modified Shannon-Wiener diversity index was used to calculate the diversity index of different traits. The calculation formula is: H′ = (−∑*P*_*i*_ln*P*_*i*_) / lnN, where H′ represents the diversity index, *P*_*i*_ is the percentage of varieties in the i_th_ grade of one trait in the total number of varieties in one period, and N is the total number of varieties in the period. The percentage of phenotypic variation explained by the population structure (*R*_Q_^2^) was analyzed using a one-way analysis of variance (ANOVA) in the SAS 9.4 system. Correlation analysis of traits was conducted by the package “corrplot” in R (3.6.0). ANOVA conducted in the EXCEL 2010 was separately used to test significant phenotypic differences between the two subpopulations. Additionally, “two sample *t*-test for means” in the SAS system was used to analyze the phenotypic or relative expression level differences between the varieties with the contrasting phenotype or haplotype.

### GWAS Analysis

Population structure analysis was conducted using the STRUCTURE software (2.3.4), and the Nei’s genetic distance’s calculation and neighbor-joining (NJ) tree construction were both conducted by PowerMarker version 3.25. Both the principle component analysis (PCA) and genome linkage disequilibrium (LD) analysis were conducted by the Tassel (5.2.51). The LD decay rate was measured as the physical distance between SNPs at which the average pairwise correlation coefficient (r^2^) went down to the half of the maximum (Huang et al. 2010). The distance matrix calculated by Tassel (5.2.51) was used to evaluate the kinship relatedness of the pairwise accessions. A total of 56,456 SNPs (full), 47,984 SNPs (Pop1), and 31,646 SNPs (Pop2) after filtering were used for association mapping according to a general linear model (GLM) using the Q matrix as the covariate and the mixed liner model (MLM) using the both PCA matrix and the kinship matrix as the covariate. Additionally, based on the Bonferroni-corrected threshold setting the effective number of independent makers at a significant level of 1, we set the *p* threshold qualification as 1/n (*n* = the number of SNP). For clustered significant SNPs within the LD decay distance of the whole genome, we chose the SNP with lowest *p* value as the lead SNP. LD block was calculated using the Haploview 4.2 to obtain the candidate regions.

### Quantitative Real-Time PCR and Sequence Analysis

Total RNA extraction from the plant’s seedling roots used the Total RNA extraction kit. First-strand cDNA was synthesized using PrimerScript RT Master Mix. Then, quantitative real-time PCR (qRT-PCR) was performed in a two-step reaction using PowerUp SYBRGreen Master Mix on an Applied Biosystems 7500 Real-Time PCR system. The rice ubiquitin gene was used as the internal control. Each measurement was performed on three replicates of each of three biological samples. The sequences of the candidate genes were downloaded from the Rice Genome Annotation Project (http://rice.plantbiology.msu.edu/analyses_search_blast.shtml). The 50 μl total PCR reaction volume contained 25 μl KOD FX PCR buffer, 10 μl dNTPs, 1.5 μl each of 10 pmol/μl primer, 5 μl template DNA, 6 μl PCR grade water, and 1 μl KOD FX. The PCR cycle conditions were as follows: initial incubation at 94 °C for 2 min, 35 cycles of 98 °C for 10 s, 55 °C for 30 s, and 68 °C at 1 kb/1 min, followed by a final extension at 68 °C for 8 min.

## Supplementary information

**Additional file 1: Figure S1.** The density of SNPs in our GWAS and phenotypic distribution in two subgroups. (a) Proportion of the 56,456 SNPs categorized by the distance to adjacent SNPs. ‘d’ represents the distance between two adjacent SNPs. (b) Boxplots of root growth ability and related traits in different subgroups. **Figure S2.** Quantile-quantile plots for 4 rooting ability-related traits in three association panels. (a) MRL in full panel. (b) RGA in full panel. (c) RL in full panel. (d) RN in full panel. (e) MRL in Pop1 panel. (f) RGA in Pop1 panel. (g) RL in Pop1 panel. (h) RN in Pop1 panel. (i) MRL in Pop2 panel. (j) RGA in Pop2 panel. (k) RL in Pop2 panel. (l) RN in Pop2 panel. Red lines represent the quantile-quantile plots of the GWAS by GLM and blue lines represent the quantile-quantile plots of GWAS by MLM. **Figure S3.** GWAS for rooting ability-related traits in the full association panel. Manhattan plots and quantile-quantile plots for MRL(a), RGA(b), RL(c), RN(d). **Figure S4.** The distribution of the significant loci on 12 chromosomes. Pink, yellow, green, blue, black, and red lines represent the loci identified from full, Pop1, Pop2, both full and Pop1, both full and Pop2, all full, and Pop1 and Pop2 association panels.

**Additional file 2: Table S1.** Summary of 145 rice accessions and their phenotypic traits related to root growth ability. **Table S2.** Significant loci detected in the full population. **Table S3.** Significant loci detected in the *indica* population. **Table S4**. Significant loci detected in the *japonica* population. **Table S5.** The primers of genes for qRT-PCR and DNA sequencing analysis. **Table S6.** The results of relative expression level of candidate genes.

## Data Availability

All data supporting the conclusions of this article are provided within the article (and its Additional files).

## Abbreviations

GWAS: Genome-wide association study
LD: Linkage disequilibrium
MRL: Maximum root length
PCA: Principle component analysis
qRT-PCR: Quantitative real-time Polymerase Chain Reaction
RGA: Root growth ability
RN: Root number
RL: Root length
SNP: Single nucleotide polymorphism
