# Supplementary material for: Genome-Wide Association Study of Rice Rooting Ability at the Seedling Stage
Source: Rice (N Y). 2020 Aug 24;13:59. doi: 10.1186/s12284-020-00420-5 (PMC7445215; doi:10.1186/s12284-020-00420-5)
Supplement: Supplementary file 1 — Additional file 1: Figure S1. The density of SNPs in our GWAS and phenotypic distribution in two subgroups. (a) Proportion of the 56,456 SNPs categorized by the distance to adjacent SNPs. ‘d’ represents the distance between two adjacent SNPs. (b) Boxplots of root growth ability and related traits in different subgroups. Figure S2. Quantile-quantile plots for 4 rooting ability-related traits in three association panels. (a) MRL in full panel. (b) RGA in full panel. (c) RL in full panel. (d) RN in full panel. (e) MRL in Pop1 panel. (f) RGA in Pop1 panel. (g) RL in Pop1 panel. (h) RN in Pop1 panel. (i) MRL in Pop2 panel. (j) RGA in Pop2 panel. (k) RL in Pop2 panel. (l) RN in Pop2 panel. Red lines represent the quantile-quantile plots of the GWAS by GLM and blue lines represent the quantile-quantile plots of GWAS by MLM. Figure S3. GWAS for rooting ability-related traits in the full association panel. Manhattan plots and quantile-quantile plots for MRL(a), RGA(b), RL(c), RN(d). Figure S4. The distribution of the significant loci on 12 chromosomes. Pink, yellow, green, blue, black, and red lines represent the loci identified from full, Pop1, Pop2, both full and Pop1, both full and Pop2, all full, and Pop1 and Pop2 association panels. [file 12284_2020_420_MOESM1_ESM.pptx]

## Slide 1
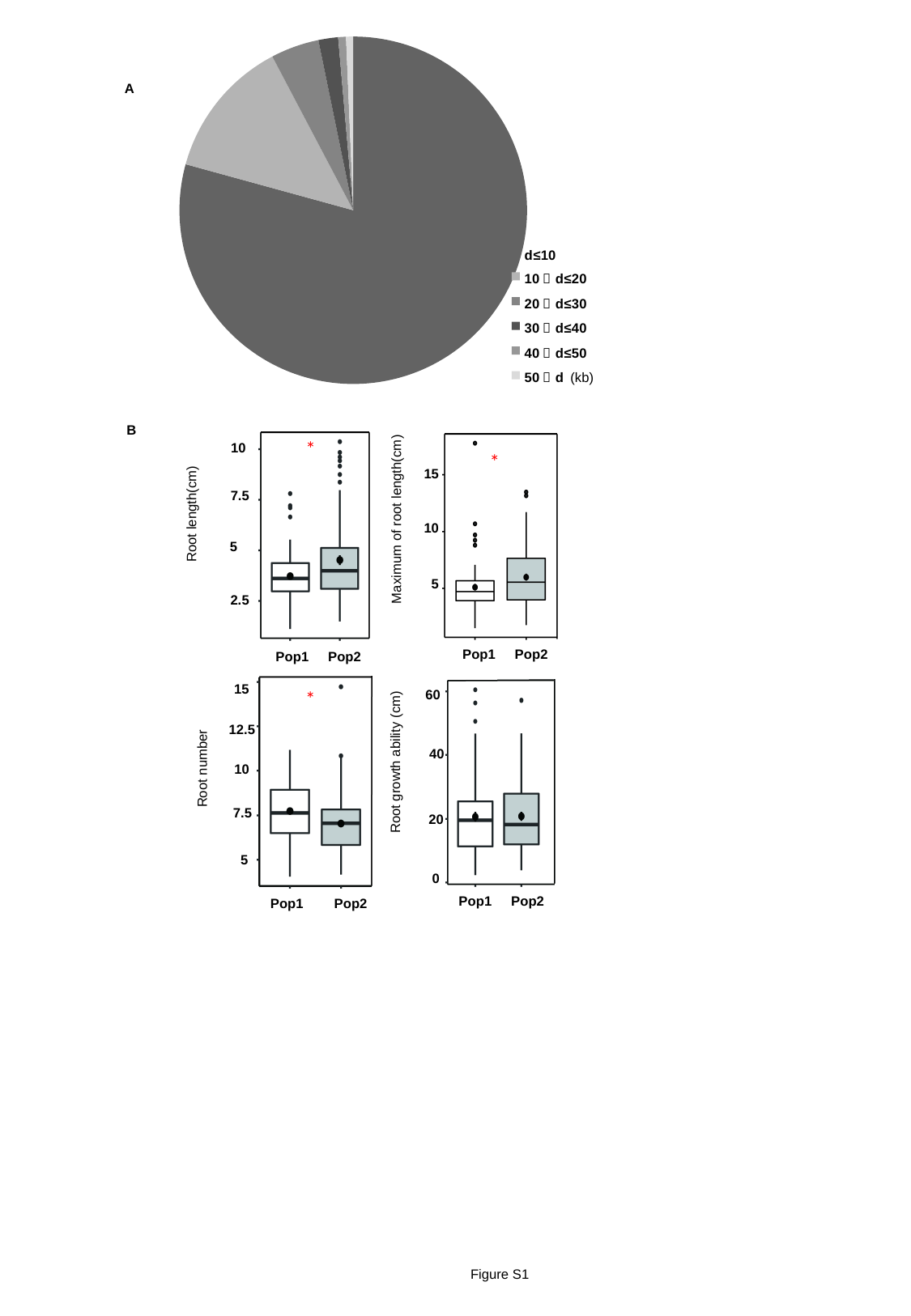

### Chart
| Category | |
|---|---|
| d≤10 | 0.7926794699170859 |
| 10＜d≤20 | 0.13023527744312946 |
| 20＜d≤30 | 0.044894054283891996 |
| 30＜d≤40 | 0.01828360853235065 |
| 40＜d≤50 | 0.007263836723123804 |
| 50＜d | 0.0066437531004181136 |(kb)
A
B
10
*
Maximum of root length(cm)
*
15
Root length(cm)
7.5
10
5
5
2.5
Pop1 Pop2
Pop1 Pop2
15
*
60
Root growth ability (cm)
40
20
0
Pop1 Pop2
12.5
Root number
10
7.5
5
Pop1 Pop2
Figure S1

## Slide 2
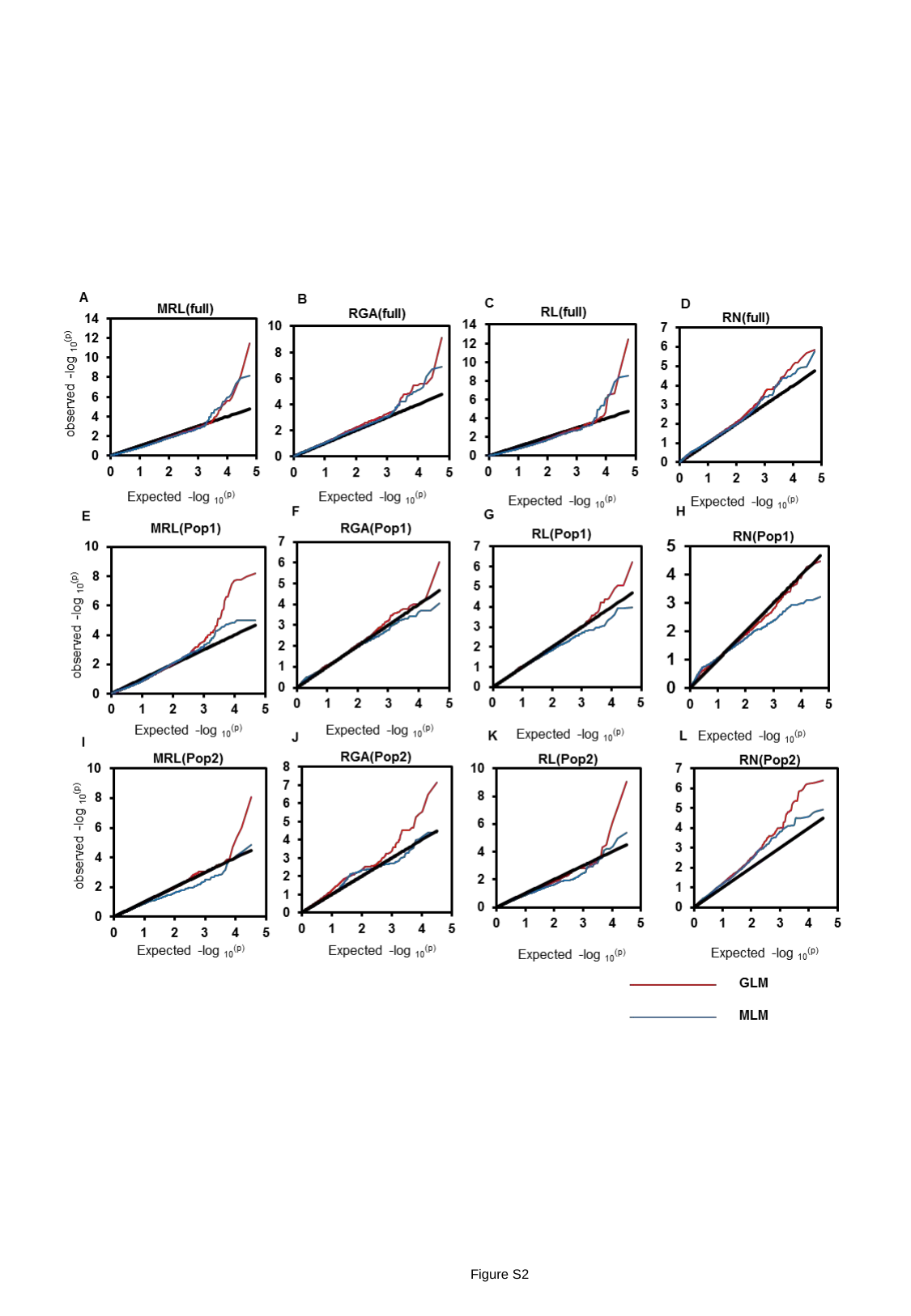

Figure S2

## Slide 3
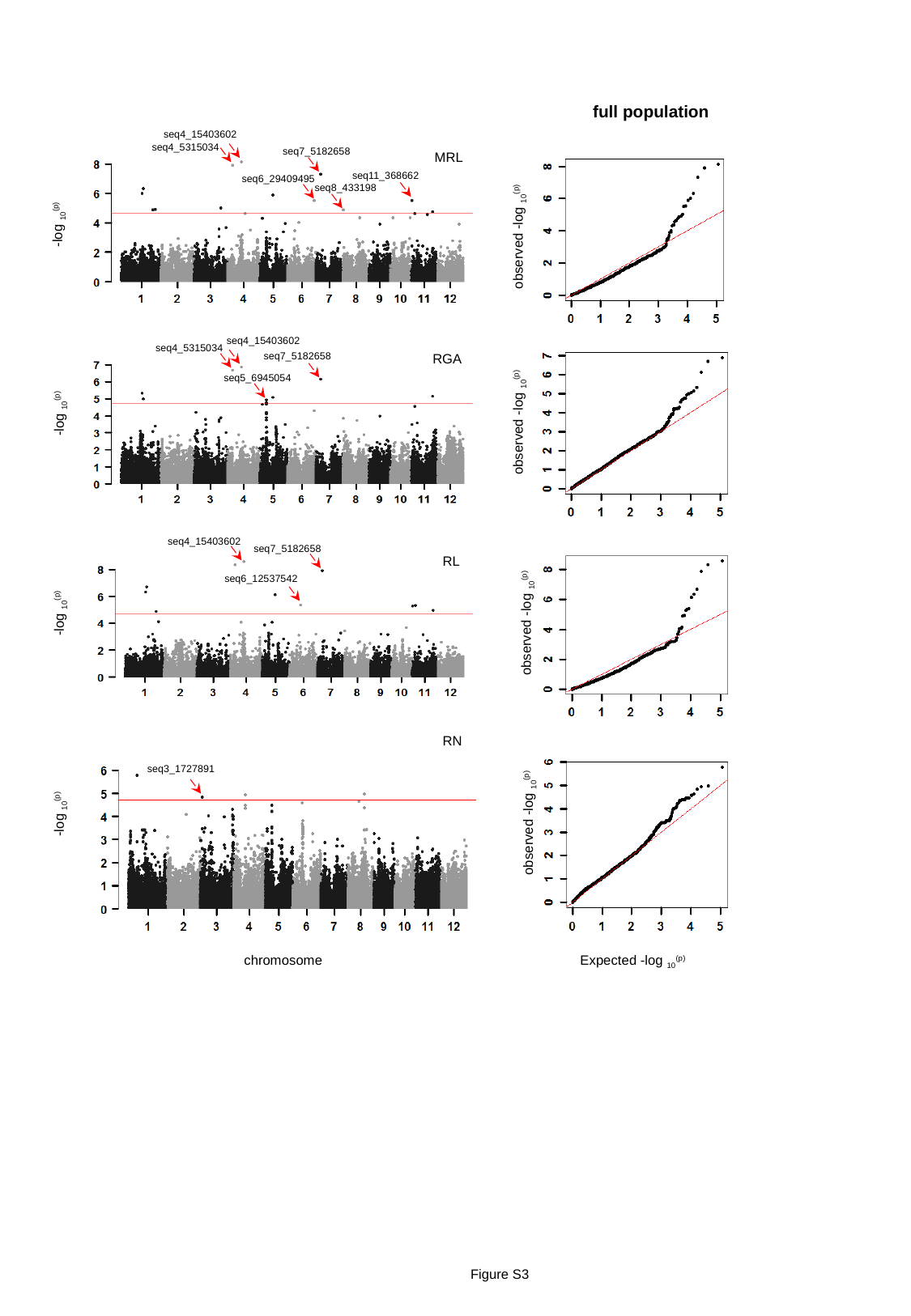

full population
seq4_15403602
seq4_5315034
seq7_5182658
MRL
seq11_368662
seq6_29409495
seq8_433198
-log 10(p)
 observed -log 10(p)
seq4_15403602
seq4_5315034
seq7_5182658
RGA
seq5_6945054
-log 10(p)
 observed -log 10(p)
seq4_15403602
seq7_5182658
RL
seq6_12537542
-log 10(p)
 observed -log 10(p)
RN
seq3_1727891
-log 10(p)
 observed -log 10(p)
chromosome
Expected -log 10(p)
Figure S3

## Slide 4
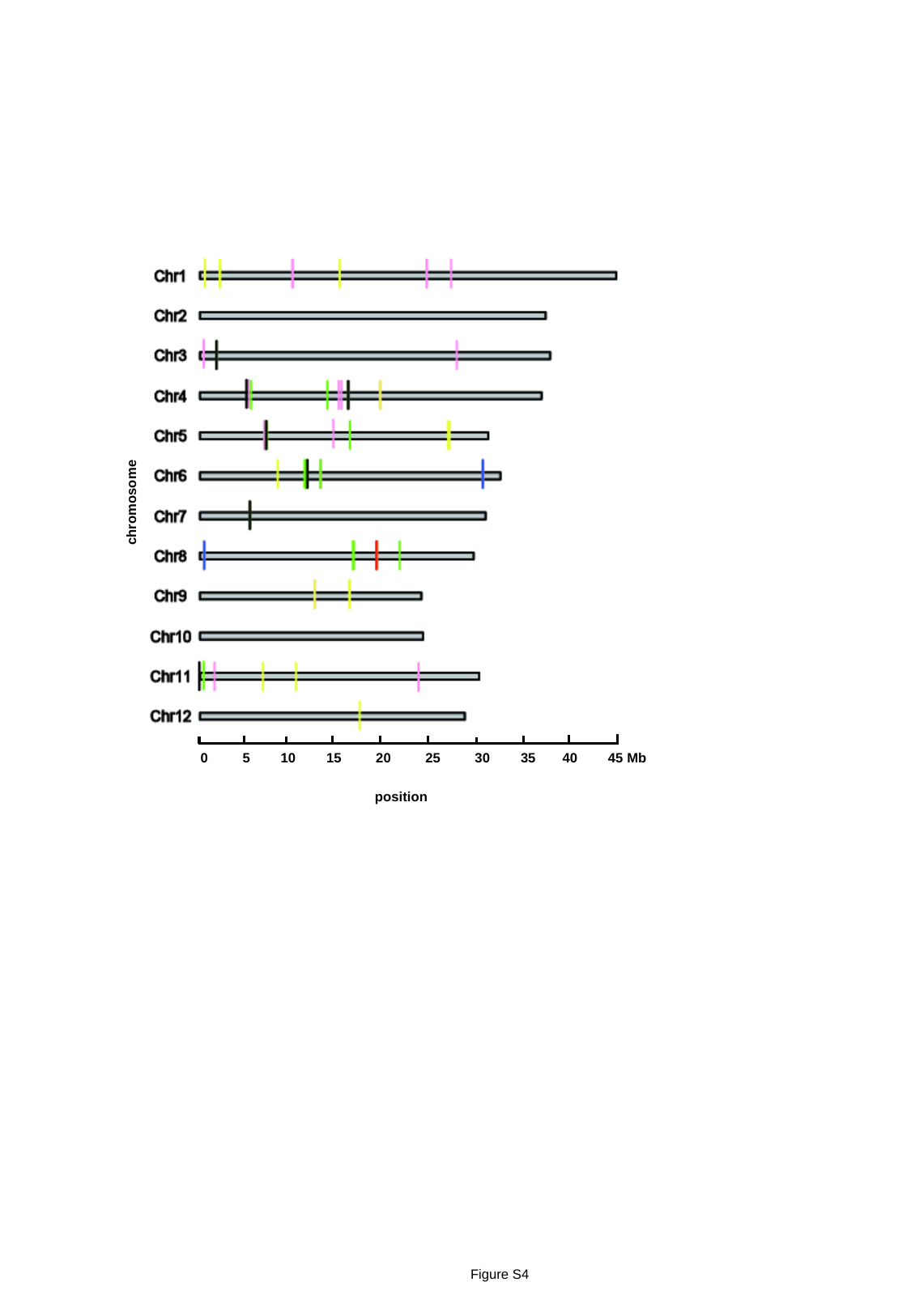

chromosome
0 5 10 15 20 25 30 35 40 45 Mb
position
Figure S4
